# Silver nanoparticles stabilized with a silicon nanocrystal shell and their antimicrobial activity

**DOI:** 10.1039/c9ra02559f

**Published:** 2019-05-15

**Authors:** Asuka Inoue, Hiroshi Sugimoto, Minoru Fujii

**Affiliations:** Department of Electrical and Electronic Engineering, Graduate School of Engineering, Kobe University Rokkodai, Nada Kobe 657-8501 Japan sugimoto@eedept.kobe-u.ac.jp fujii@eedept.kobe-u.ac.jp

## Abstract

The antimicrobial activity of a hybrid nanoparticle (NP) composed of a silver (Ag) NP core decorated with silicon (Si) nanocrystals (NCs) on the exterior (Ag/Si NPs) is evaluated. The shell of Si NCs effectively protects the surface of Ag NPs, thus the particles are more stable in water and in air compared to conventional organic-capped Ag NPs. The bacterial growth kinetic analysis reveals that the Si NC shell does not suppress the release of Ag ions from the Ag NP surface due probably to the porous structure. For the antimicrobial coating application, a thin film of the hybrid Ag/Si NPs is produced by drop coating the solution on a cover glass. Thanks to the Si NC shell, agglomeration of Ag NPs in the film is prevented and the film shows a very similar optical absorption spectrum to that of the solution. The film exhibits a larger zone of inhibition in an agar diffusion assay of *Escherichia coli* compared to a film produced from organic-capped Ag NPs.

## Abstract 2

The antimicrobial activity of a hybrid nanoparticle (NP) composed of a silver (Ag) NP core decorated with silicon (Si) nanocrystals (NCs) on the exterior (Ag/Si NPs) is evaluated.

## Graphical abstract

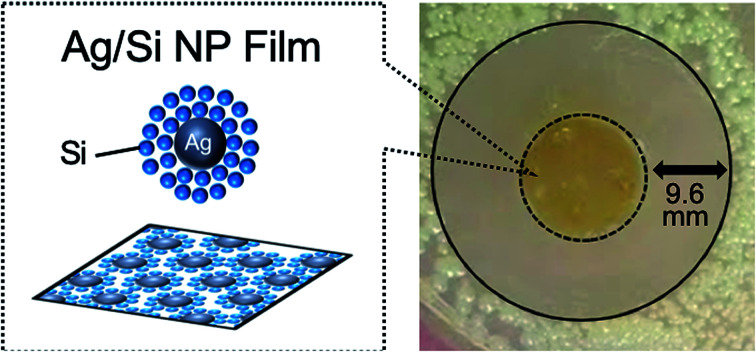

## Introduction

With the increase in bacterial mutations and resistance development against small-molecule antibiotics, there is a strong demand to develop antimicrobial agents with low potential for mutations, while displaying broad-spectrum biocidal activity.^1–4^ Silver (Ag) nanoparticles (NPs) are one of the most widely studied antimicrobial nanomaterials due to their potential to suppress the growth of bacteria by the release of Ag ions, especially against Gram negative bacteria such as *Escherichia coli* (*E. coli*).^5–7^ Bacterial resistance against Ag-based antibiotics is in general very small and the true mutational development of resistance against Ag ions is relatively uncommon compared to small molecule antibiotics.^8^ In Ag NPs, the large surface-to-volume ratio compared to other Ag-based antimicrobial materials makes the release of Ag ions more efficient, resulting in the high antimicrobial activity while maintaining the low tissue cytotoxicity.^9–14^

One of the drawbacks of Ag NPs as an antimicrobial agent is the relatively low physicochemical stability due to the oxidization and the agglomeration. In order to achieve the high physicochemical stability while maintaining the high antimicrobial activity, composites with different materials have been proposed.^15–23^ Jain *et al.* developed an antimicrobial water filter by embedding Ag NPs in a polyurethane filter for the suppression of bacteria growth in water.^15^ Lv *et al.* developed a Ag-NP-decorated silicon (Si) nanowire and demonstrated the stable antimicrobial effect against *E. coli*.^16^ Kumar *et al.* produced an antimicrobial paint by embedding Ag NPs in vegetable oil and demonstrated the antimicrobial effects on both Gram positive and negative bacteria.^17^

In this work, we propose a new Ag-NP-based composite NPs as an antimicrobial agent. The composite NP is composed of a Ag NP core and a thin shell made of a few layers of Si nanocrystals (NCs) about 3 nm in diameter. The structure, *i.e.*, the core size and the shell thickness, of the composite NP can be controlled in wide ranges.^24^ This allows us to simultaneously achieve the high physicochemical stability and the high antimicrobial activity. Since the surface of the Si NCs is negatively charged (zeta potential: −35 mV (ref. 25)), agglomeration of the composite NPs in aqueous media is perfectly prevented by the electrostatic repulsion: the composite NPs are dispersed in aqueous media without organic ligands. Furthermore, because of the thin Si NCs coating, agglomeration of Ag NPs is prevented even in a film made from the solution. Therefore, a uniform film of Ag NPs, whose optical absorption spectrum is very similar to that of the solution, can be produced. We show that the film exhibits a larger zone of inhibition in an agar diffusion assay of *E. coli* compared to a film produced from organic-capped Ag NPs.

## Results and discussion

### Preparation of silver nanoparticles stabilized with a silicon nanocrystal shell

Ag/Si NPs were prepared by the procedure described in ref. 24. First, a methanol solution of boron (B) and phosphorus (P) codoped Si NCs were prepared by a cosputtering method. Details of the preparation procedure are shown in our previous paper.^26^ The codoped Si NCs have heavily B and P codoped amorphous shell.^27,28^ The shell induces negative potential on the surface of Si NCs and prevents the agglomeration in polar solvents. Therefore, the codoped Si NCs are perfectly dispersed in methanol for years without any organic functionalization processes.^29^ Furthermore, thanks to the shell, the codoped Si NCs have high resistance to oxidation and exhibit stable photoluminescence not only in methanol but also in water.^25^ Although hydrogen (H) atoms on the outer surface of codoped Si NCs are slowly replaced by oxygen atoms during the storage in methanol, oxidation does not proceed further. Throughout this work, we use a freshly prepared H-terminated codoped Si NCs for the preparation of Ag/Si NPs.

**Fig. 1:**
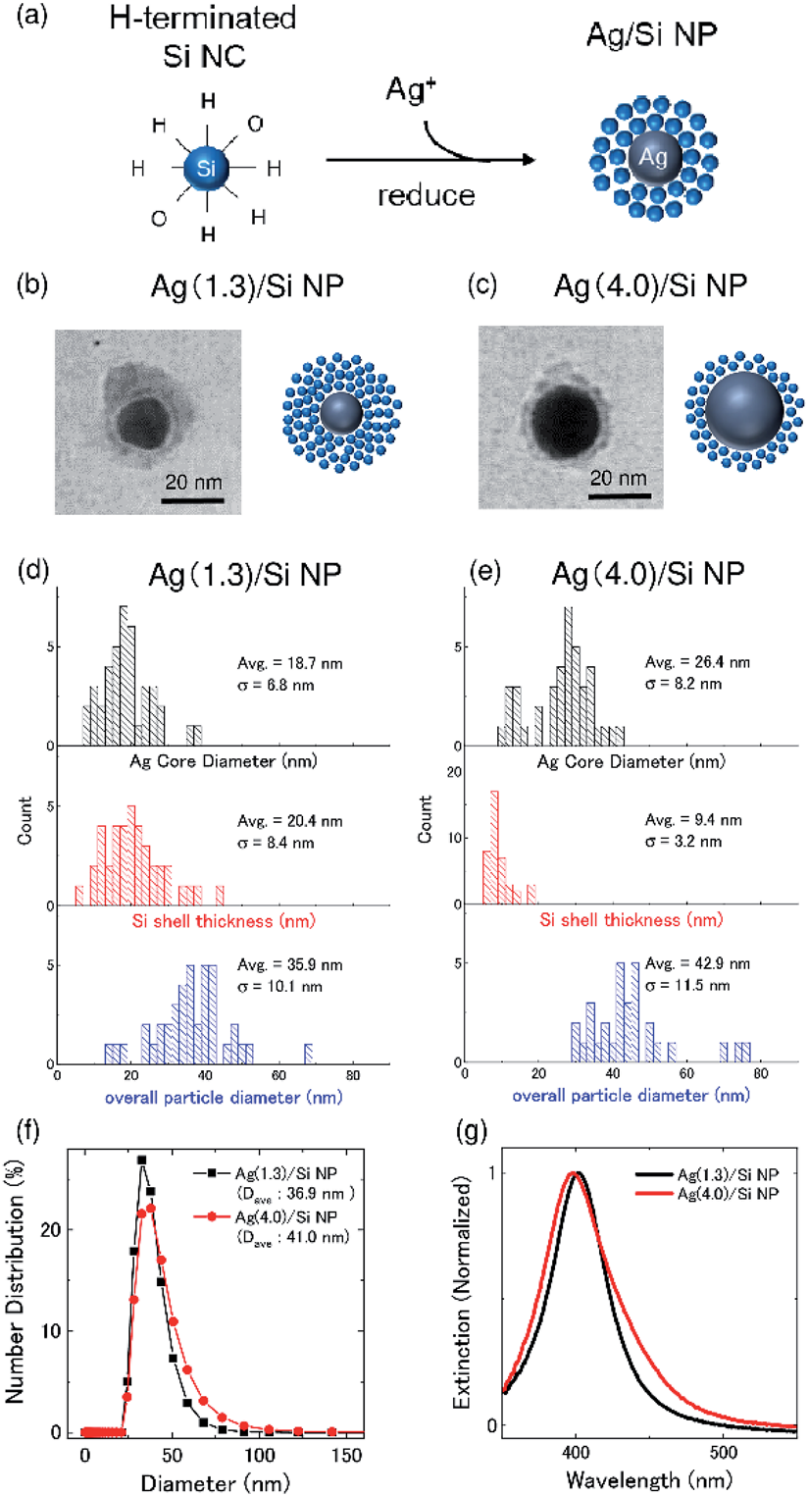
(a) Schematic depicting of the preparation process of Ag/Si NPs, which uses Si NCs as a combination of reducing and capping agent. (b and c) TEM images of Ag(1.3)/Si NPs (b) and Ag(4.0)/Si NPs (c). (d and e) Histograms from TEM measurements showing Ag NP core diameter (black, top), Si NC shell thickness (red, middle) and overall particle diameter (blue, bottom) of Ag/Si NPs. (d) Ag(1.3)/Si NPs; (e) Ag(4.0)/Si NPs. (f) Size distributions of Ag/Si NPs measured by DLS. (g) Absorption spectra of aqueous dispersions of Ag(1.3)/Si NPs (black trace) and Ag(4.0)/Si NPs (red trace).

Ag/Si NPs were prepared by a self-limiting growth process similar to the well-known Turkevich method^30^ by using H-terminated codoped Si NCs as a reducing agent of silver nitrate (AgNO_3_) and also as a protective layer to stop the growth of Ag NPs as schematically shown in Fig. 1a.^24^ Details of the preparation procedure and the characterization results are found in our previous paper.^24^ The advantage of this method is that the size of a Ag core and the thickness of a Si NCs shell can be controlled in a wide range by controlling the preparation parameters.^24^ Furthermore, because of the hydrophilic nature of codoped Si NCs, the Ag/Si NPs are dispersed in polar solvents without any additional processes. In the actual preparation procedure, a 580 μL methanol solution of Si NCs (0.1 mg mL^−1^) are mixed with a 20 μL methanol solution of AgNO_3_ under vigorous stirring at room temperature.^24^ The AgNO_3_ concentration (Ag^+^ concentration) in mixed solutions is 1.3 and 4.0 mM, which corresponds to the Ag metal concentration of 140 and 430 μg mL^−1^, respectively. Hereafter, we denote Ag/Si NPs prepared with the AgNO_3_ concentration of 1.3 and 4.0 mM as Ag(1.3)/Si NPs and Ag(4.0)Si NPs, respectively.

Fig. 1b and c shows the representative TEM images of Ag(1.3)/Si NPs and Ag(4.0)/Si NPs, respectively. High-resolution TEM observations and energy dispersive X-ray spectroscopy (EDS) in a previous study revealed that the NPs are composed of a Ag NP core and a shell made from Si NCs.^24^Fig. 1d and e shows the Ag core diameter (black), Si shell thickness (red), and overall particle diameter (blue) of Ag/Si NPs. The average diameter of a Ag core increases from 18.7 nm to 26.4 nm, when AgNO_3_ concentration increases from 1.3 mM to 4.0 mM, while the shell thickness decreases from 20.4 to 9.4 nm. The decrease of the shell thickness is due to fixed supply of Si NCs in the Ag/Si NPs growth process. Considering the diameter of Si NCs (∼3 nm in diameter),^29^ the shells are composed of approximately 7 and 3 layers of Si NCs for Ag(1.3)/Si NPs and Ag(4.0)/Si NPs, respectively. The average overall diameters of Ag/Si NPs are 35.9 nm and 42.9 nm for Ag(1.3)/Si NPs and Ag(4.0)/Si NPs, respectively. These values are close to the average hydrodynamic diameters obtained from dynamic light scattering (DLS) shown in Fig. 1f. Fig. 1g shows extinction spectra of Ag/Si NPs in methanol. The peak around 400 nm is due to localized surface plasmon resonances (LSPRs) of Ag NPs.

**Fig. 2:**
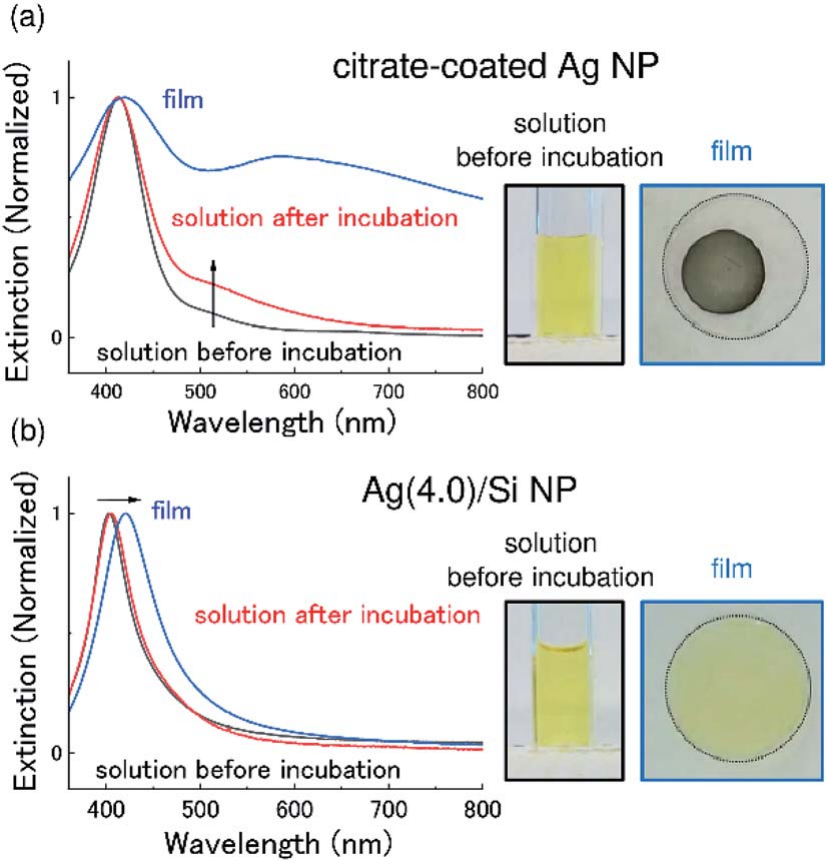
Absorption (extinction) spectra of an aqueous dispersion of (a) citrate-coated Ag NPs and (b) Ag(4.0)/Si NPs. Black and red traces are solution samples before and after incubation at 37 °C for 15 days, respectively, and blue traces represent a dry film samples produced from the solutions. Photographs of samples are shown on the right (solutions are before incubation).

As mentioned in the introduction, stability is an important issue for antimicrobial applications of Ag NPs. Fig. 2a shows the absorption (extinction) spectra of commercially available citrate-coated Ag NPs (40 nm in diameter) before and after incubation at 37 °C for 15 days. Before the experiments, citrate-coated Ag NPs were washed and re-suspended in water. After the incubation, the LSPR peak becomes broad and a tail appears in the longer wavelength range. It is well-known that agglomeration of Ag NPs leads to the coupling of LSPRs, which results in the broadening and the red shift of the absorption spectra.^31,32^ The observed change in the spectral shape thus suggests that agglomeration proceeds during the incubation.

In Fig. 2b, the results of the same experiments performed for Ag/Si NPs are shown. Similar to the case of citrate-coated Ag NPs, the Ag/Si NPs were washed and re-suspended in water before the experiments. We can see that the absorption spectrum after the incubation is almost identical to that before the incubation. This result confirms that a Si NC shell effectively prevents agglomeration of Ag NPs and provides the high colloidal stability.

In Fig. 2a and b, pictures of colloidal solutions and films produced by drop coating the solutions are also shown. In citrate-coated Ag NPs (Fig. 2a), in contrast to the clear yellowish color of the solution, the film is dark gray. The change of the color can be seen also in the extinction spectrum as a significant broadening of the LSPR peak. The change of the LSPR behavior is due to agglomeration and oxidation of Ag NPs by exposure to air for the film formation. Similar significant broadening of the LSPR is observed when a film is produced from as-purchased (not washed) citrate-coated Ag NPs.

In contrast to citrate-coated Ag NPs, the color of the film produced from a Ag/Si NPs solution (Fig. 2b) is very similar to that of the solution and the change of the absorption (extinction) spectrum is very small. The slight long wavelength shift of the LSPR peak is explained by the increase in the effective dielectric constant around the Ag NPs due to higher density of Ag NPs in a film than in a solution. It is worth noting that the film maintains the color for more than 3 months.

### Antimicrobial activity of Ag/Si NPs on *E. coli* in LB broth

**Fig. 3:**
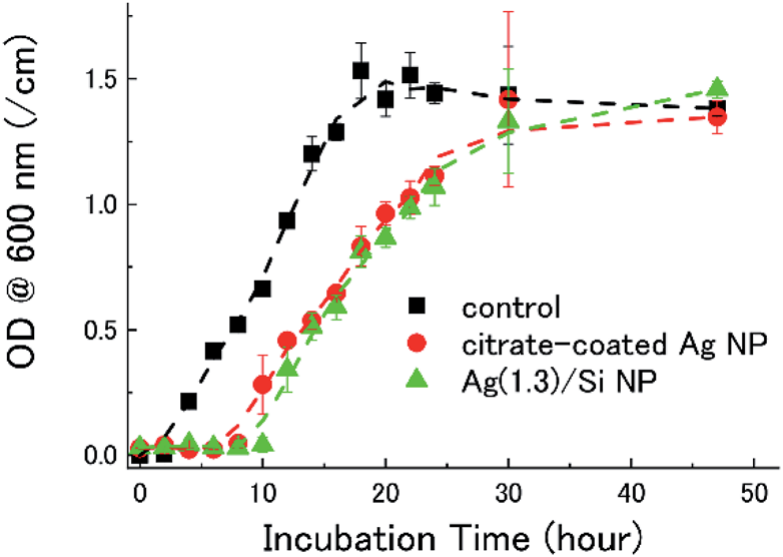
Growth curves of *E. coli* bacteria in LB broth containing citrate-coated Ag NPs and Ag(1.3)/Si NPs. The quantity of Ag atoms in the bacterial broths is the same (2 μg mL^−1^). Control represents bacterial culture without any Ag formulations (black). Bacterial growth is measured as the optical density at 600 nm. Incubation time = 0 h corresponds to the time at which the relevant nanomaterial is added to the bacterial broth. For each condition, the experiments are performed three times and the average is plotted.

Fig. 3 shows the growth curves of *E. coli* in a LB broth containing citrate-coated Ag NPs and Ag/Si NPs. In the control data (black), *i.e.*, a bacterial culture without Ag NPs, the lag phase of the *E. coli* growth curve lasts 2 hours. When citrate-coated Ag NPs (2 μg mL^−1^) are co-incubated (red), the lag phase lasts 8 hours. Similarly, when Ag(1.3)/Si NPs are co-incubated (green), the lag phase is 10 hours. Note that the quantity of Ag atoms in the bacterial broths is the same (2 μg mL^−1^) in both experiments. Thus, the Si NCs shell, which stabilizes Ag NPs in water and in air, does not degrade the antimicrobial activity of Ag NPs. The bacterial growth is perfectly suppressed when the concentration of Ag(1.3)/Si NPs is 14 μg mL^−1^ (data not shown).

### Antimicrobial activity of Ag/Si NP films on *E. coli* in agar diffusion assay

For antimicrobial coating applications, we prepare films of Ag/Si NPs by drop coating the aqueous solutions (40 μL) onto a circular cover glass (12 mm in diameter) and drying in air for 1 h and in a vacuum desiccator for 2 h. As references, we also prepared films from citrate-coated Ag NPs by exactly the same process.

**Fig. 4:**
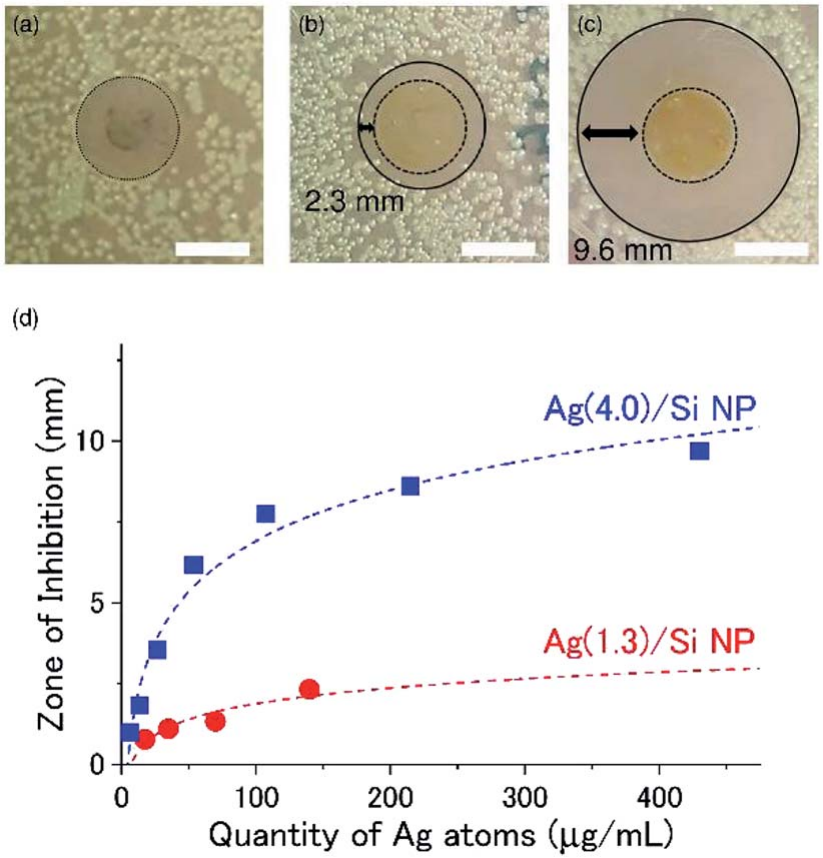
Zone of inhibition tests of antimicrobial films prepared by drop coating 40 μL of (a) citrate-coated Ag NPs solution (Ag concentration: 430 μg mL^−1^), (b) Ag(1.3)/Si NPs (Ag concentration: 140 μg mL^−1^) solution, and (c) Ag(4.0)/Si NPs (Ag concentration: 430 μg mL^−1^) solution. Circular glass cover slips (12 mm diameter) with the films are placed face-down on the growth media containing the *E. coli* cultures. The white scale bars show 10 mm. The images are annotated with a dashed black circle to indicate the circumference of the cover glass containing NPs, and a solid black circle indicates the demarcation between regions of low and high bacterial growth, *i.e.*, the zone of bacterial growth inhibition around the cover glass/NP film. The inhibition zone is defined as the difference between the radius of the solid black circle and the dashed black circle. Images are obtained 24 h after introduction of the NPs. (d) The size of the inhibition zone as a function of the quantity of Ag atoms on the cover glass. Red and blue dashed lines indicate results of fittings by eqn (2). For each condition, the experiments are performed three times and the average is plotted. The variation of the data (error bars) are smaller than the size of the symbol.

Fig. 4a shows a picture of an LB agar plate on which a Ag NP film produced from a citrate-coated Ag NP is placed after incubation at 37 °C for 24 h. We can see no discernible zone of inhibition around the film. On the other hand, Ag/Si NP films have wide inhibition zones. In the Ag(1.3)/Si NP film (Fig. 4b), the inhibition zone, which is defined by the distance from the edge of a film to the end of the bacteria clearance zone, is about 2.3 mm, while in the Ag(4.0)/Si NP film (Fig. 4c), it is about 9.6 mm.

We performed the same experiments by changing the Ag/Si NP concentration in a solution for the preparation of the films in a wide range. Fig. 4d shows the size of the inhibition zone as a function of the Ag concentration. The abscissa can be converted to the quantity of Ag atoms in films by multiplying 40 μL. The zone of inhibition depends not only on the quantity of Ag atoms but also on the structure of Ag/Si NPs. Ag(4.0)/Si NPs has a larger inhibition zone than Ag(1.3)/Si NPs.

In order to quantitatively discuss the observed relation between the inhibition zone size and the Ag concentration, we adopt a widely used absorptive diffusion model.^33^ In the model, the antibiotic concentration, *c*(*x*,*t*), can be expressed as,1
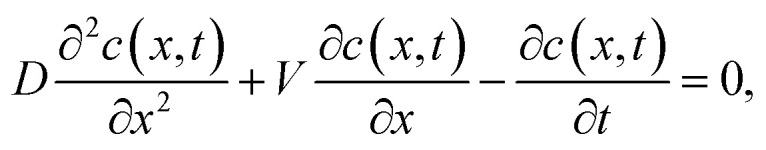
where *x* is the distance from the source, *t* is the incubation time, *D* is the diffusion coefficient, and *V* is the coefficient characterizing the dissipation rate. The general form of the space part of the solution shows diffusion as absorption dominated and exponentially decaying. For the case of semi-infinite medium, the solution is expressed as,^33^2
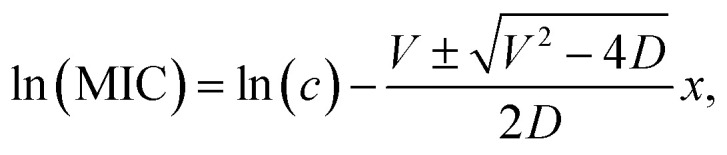
where MIC is the minimum inhibitory concentration of antibiotics. Dashed curves in Fig. 4d are the results of the fitting of the experimental data with eqn (2) by using MIC, *D* and *V* as fitting parameters. The experimental data can be well reproduced by the model. The fitting parameters are summarized in Table 1. The MIC of Ag(4.0)/Si NPs is 4.7 μg mL^−1^, which is smaller than that of Ag(1.3)/Si NPs, *i.e.*, 7.1 μg mL^−1^. This is consistent with the larger inhibition zone in Ag(4.0)/Si NPs than in Ag(1.3)/Si NPs in Fig. 4d. It may be possible that the thinner Si NCs shell in Ag(4.0)/Si NPs than in Ag(1.3)/Si NPs is responsible for the larger antimicrobial activity.

**Table 1:** Minimum inhibitory concentration of antibiotics (MIC), diffusion coefficient (*D*) and dissipation coefficient (*V*) obtained by fitting the data in Fig. 4d with eqn (2), and calculated viscosity

	Ag(1.3)/Si NP	Ag(4.0)/Si NP
MIC (μg mL^−1^)	7.1	4.7
*D* (m^2^ s^−1^)	6.8 × 10^−12^	3.5 × 10^−12^
*V* (m s^−1^)	3.1 × 10^−8^	2.0 × 10^−8^
Viscosity (mPa s)	0.91	1.59

For a spherical particle of radius *r* in a solvent of viscosity *η*, the diffusion coefficient *D* is given by3
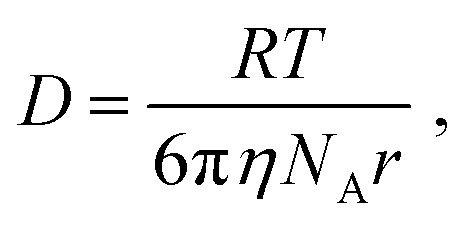
where *R* is the gas constant, *N*_A_ is the Avogadro's number, and *T* is the absolute temperature. Inserting the value of *D* obtained from the fitting and the hydrodynamic diameter into eqn (3), the viscosity is obtained. They are 0.91 and 1.59 mPa s in Ag(1.3)/Si NPs and Ag(4.0)/Si NPs, respectively. These values are close to the viscosity of water (∼1.00 mPa s). This suggests that Ag diffuses from the film to condensed water on the surface of agar. However, at this stage of research, the form of the diffusing objects is not very clear. A possible model is that Ag/Si NPs diffuse into the Petri dish from the film and finally dissolves to generate Ag^+^ ions at the place far from the edge of the film, although further researches are necessary to prove the hypothesis.

## Conclusions

Antimicrobial activity of a hybrid NP composed of a Ag NP core decorated with hydrophilic Si NCs on the exterior is studied. The shell of Si NCs effectively protects the surface of Ag NPs, and thus the particles are more stable in water and in air compared to conventional organic-capped Ag NPs. Despite the effective protection of a Ag NP core, Si NC shell does not degrade the release rate of Ag ions. Furthermore, thanks to the Si NC shell, agglomeration of Ag NPs is prevented even in a film produced by drop coating the solution. The film exhibits a much larger zone of inhibition in an agar diffusion assay of *E. coli* compared to a film produced from organic-capped Ag NPs.

## Experimental section

### Materials

Citrate-coated Ag NPs (40 nm in diameter, Cat No. 730807) were purchased from Sigma Aldrich. The silver nitrate (AgNO_3_) was purchased from FUJIFILM Wako Pure Chemical Corporation. A non-pathogenic *E. coli* strain (FDA strain Seattle 1946) was obtained from American Type Culture Collection (ATCC). The Luria–Bertani (LB) broth and LB agar plate for bacteria culture were purchased from TEKnova.

### Preparation of colloidal solution of B and P codoped Si NCs

Si, SiO_2_, B_2_O_3_, and P_2_O_5_ were simultaneously sputtered, and a Si rich borophosphosilicate glass (BPSG) film was deposited on a stainless steel plate. The film was peeled off from the plate and annealed in a N_2_ gas atmosphere at 1100 °C for 30 min to grow codoped Si NCs in a BPSG matrix. In this growth condition, Si NCs about 3 nm in diameter are grown. Si NCs were then extracted from a matrix by hydrofluoric acid (HF) etching. Finally, Si NCs in a HF solution were transferred to methanol. Details of the preparation procedure and the characterization results are shown in our previous papers.^34,35^

### Characterization of Ag core/Si NCs shell hybrid NP

The mean hydrodynamic diameter of Ag/Si NPs were measured by DLS (Malvern Zetasizer Nano ZS90). The structure of Ag/Si NPs were studied by TEM (HITACHI H-7000). Histograms of a Si NC shell thickness, a Ag core diameter and an overall particle diameter were obtained from TEM images of 40 particles. Light absorption (extinction) spectra of a solution and a film were obtained from the transmittance spectra measured by a spectrophotometer (Shimadzu UV-3101PC).

### Bacterial culture

*E. coli* was cultured in a LB broth and incubated in a shaking incubator overnight at 37 °C and at 59 rpm (GYROMAC 737) (Amerex Instruments, Inc.). The culture was diluted to 10^8^ CFU mL^−1^, corresponding to an optical density at 600 nm (OD600) of 0.1.

### *In vitro E. coli* growth in suspension

For *in vitro* growth assay, *E. coli* suspensions with or without NPs were dispersed in a LB broth at a concentration of 10^6^ CFU mL^−1^, then incubated for 48 h in a shaking incubator at 37 °C and at 59 rpm (GYROMAC 737) (Amerex Instruments, Inc.). OD at 600 nm was measured in 2 hour intervals until 24 h, and then at 30 and 48 h. Prior to the assay, both citrate-coated Ag NPs and Ag/Si NPs were washed twice by a LB broth by centrifugation for 10 min at 10 000*g* at 20 °C.

### Agar diffusion assay

A 100 μL suspension of *E. coli* (10^5^ CFU mL^−1^ in LB broth) was spread onto an LB agar plate. A Ag/Si NP film on a cover glass was placed on the agar plate face-down, such that the Ag/Si NPs were in direct contact with the agar plate. The plate was incubated for 24 hours at 37 °C in an incubator (Heratherm) (Thermo Fisher Scientific). The zone of inhibition was defined as a distance from the edge of the cover glass to the edge of the bacteria clearance zone.

## Conflicts of interest

There are no conflicts to declare.

## Supplementary Material
